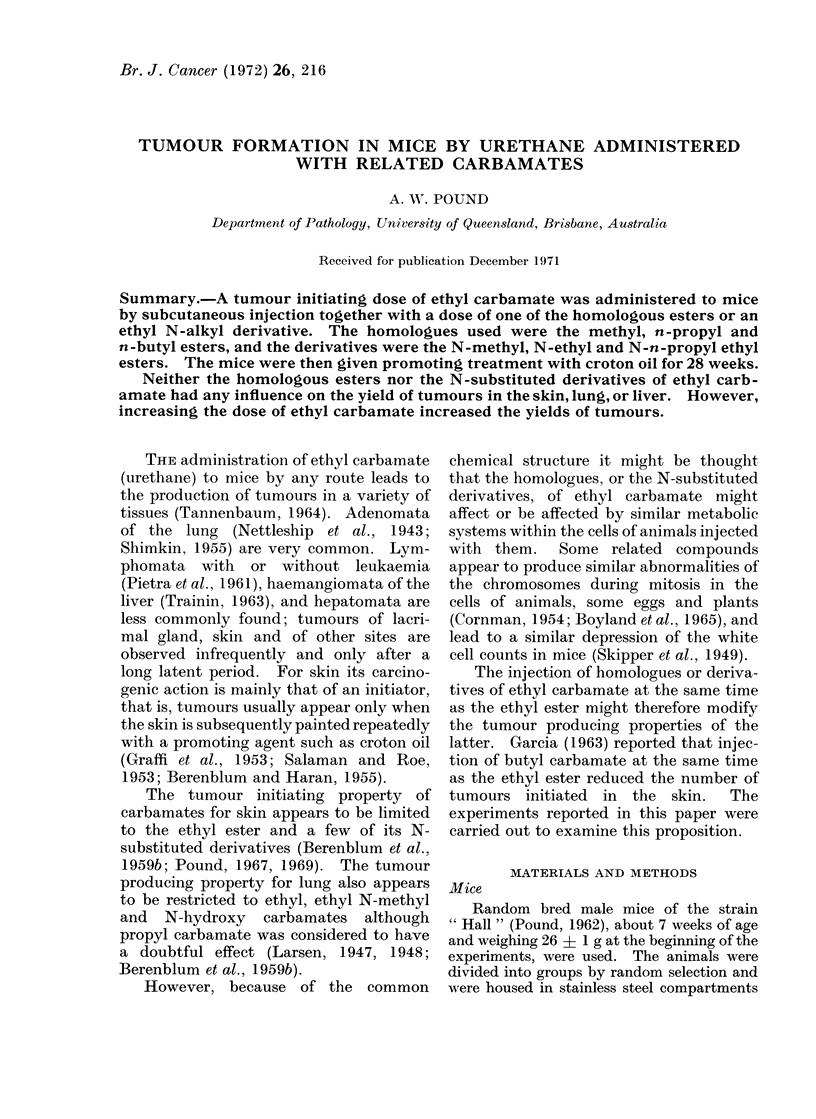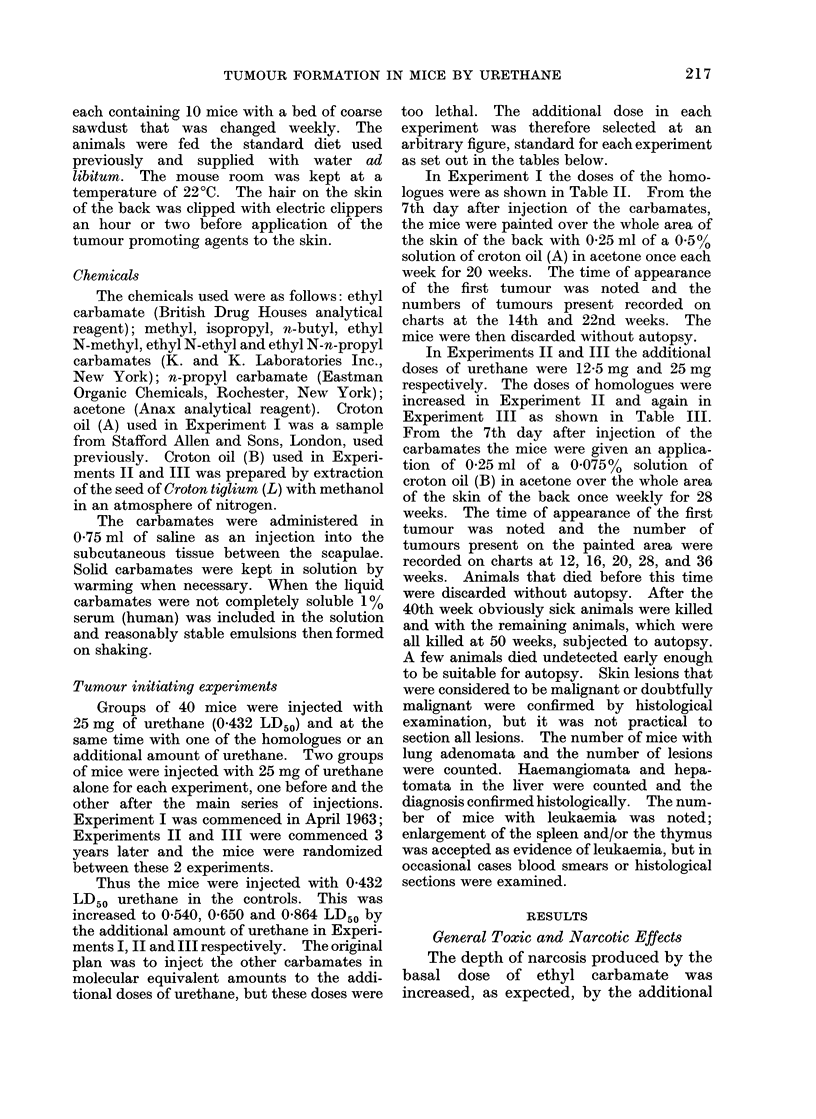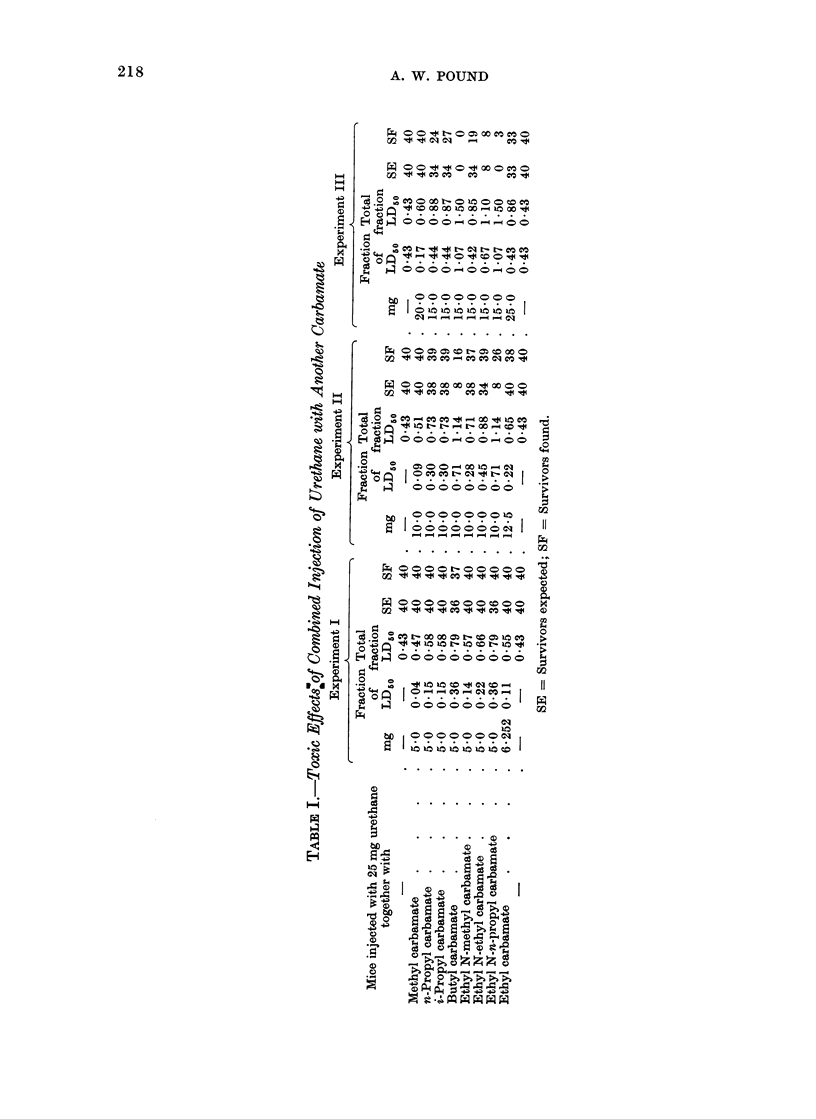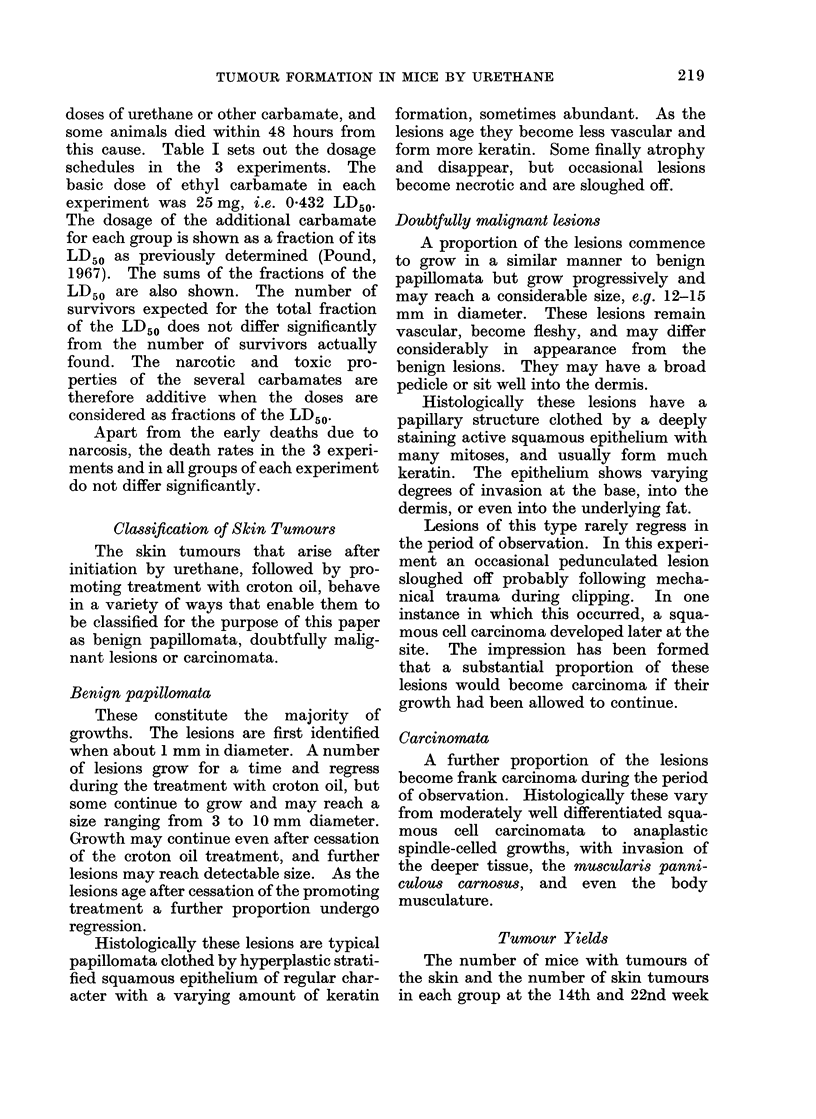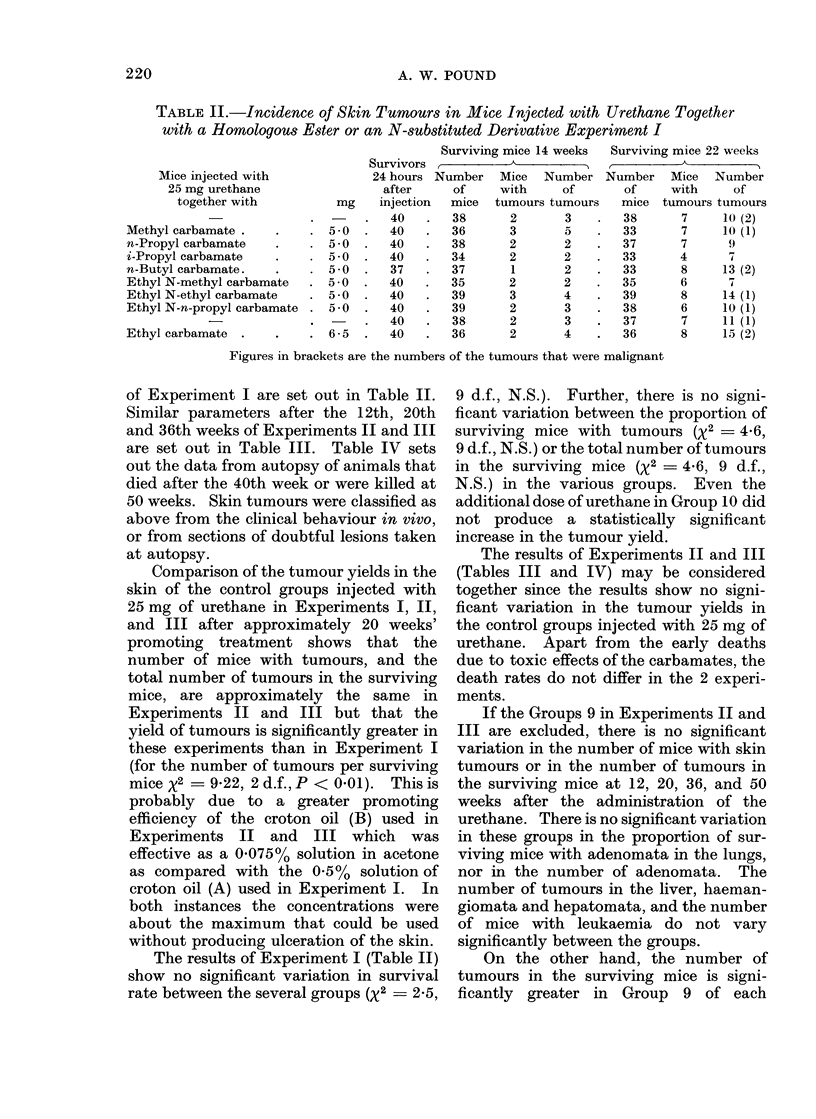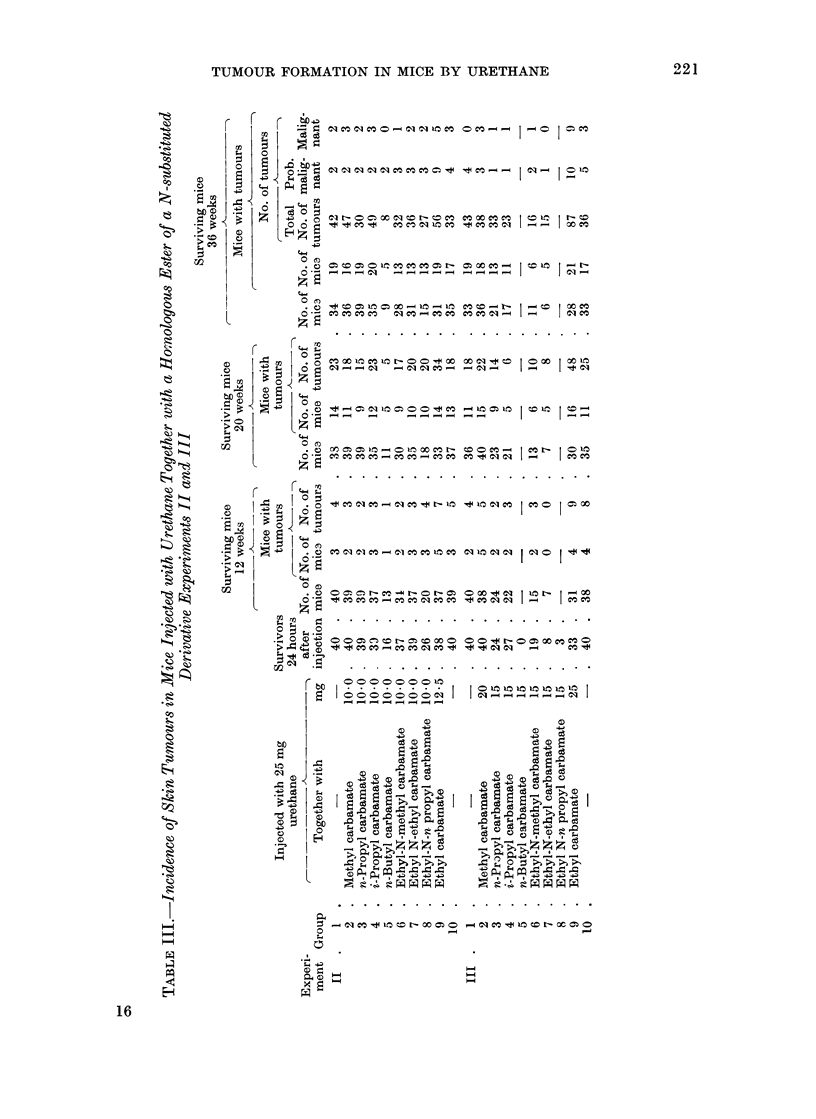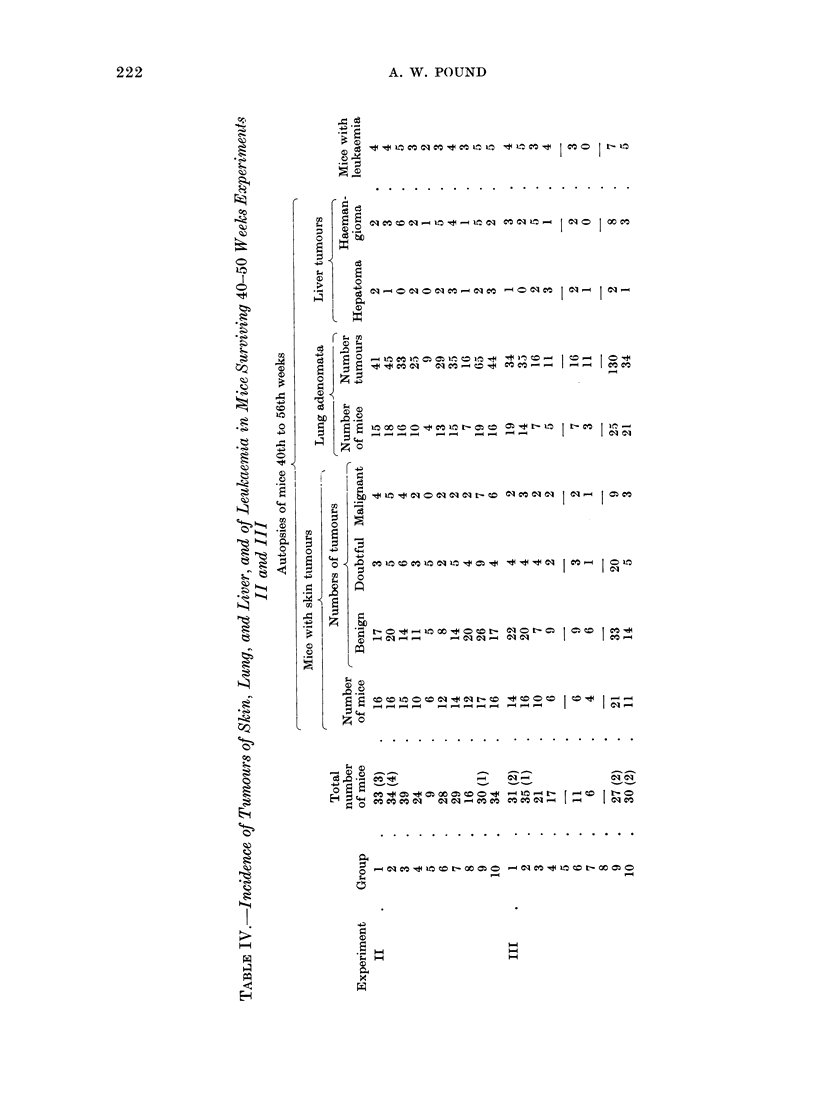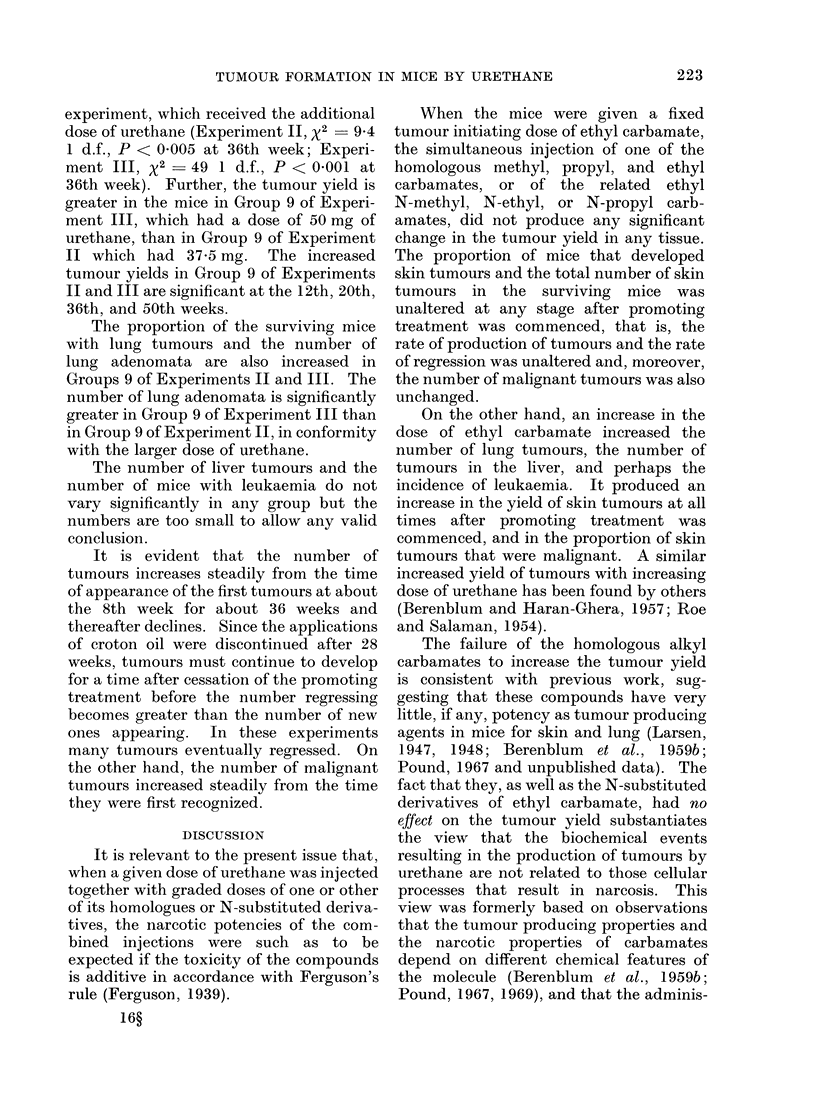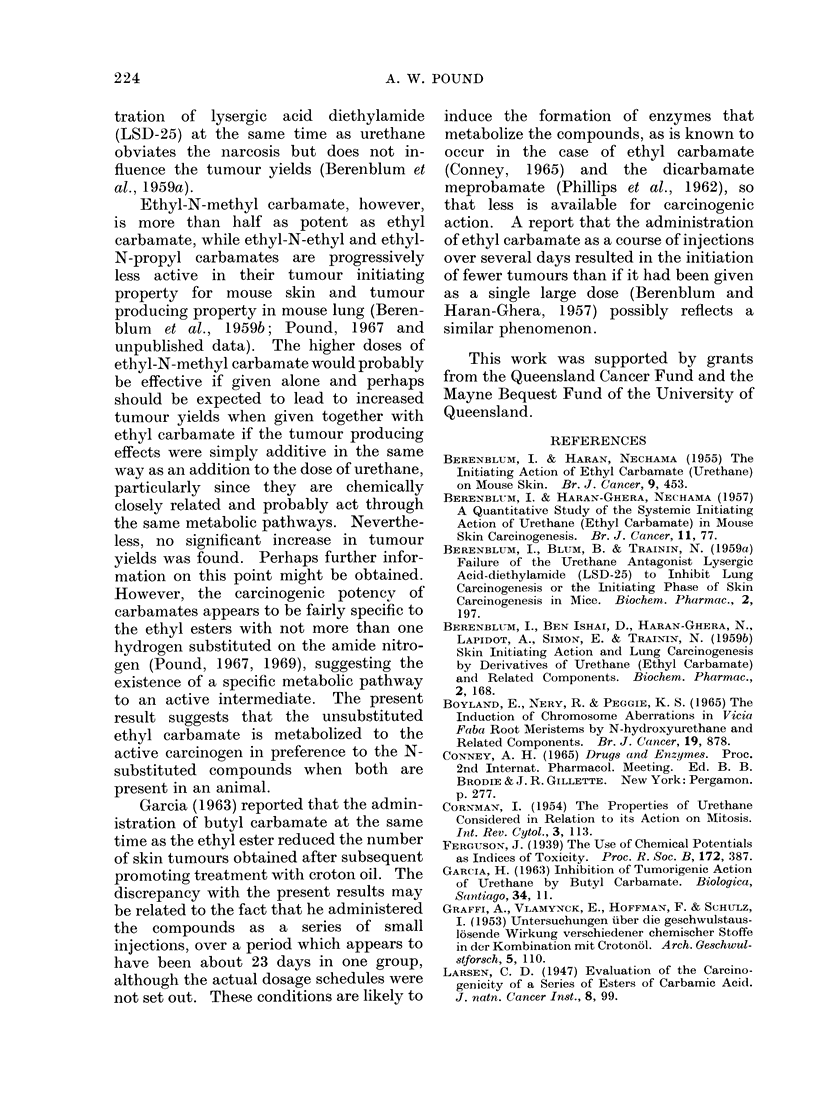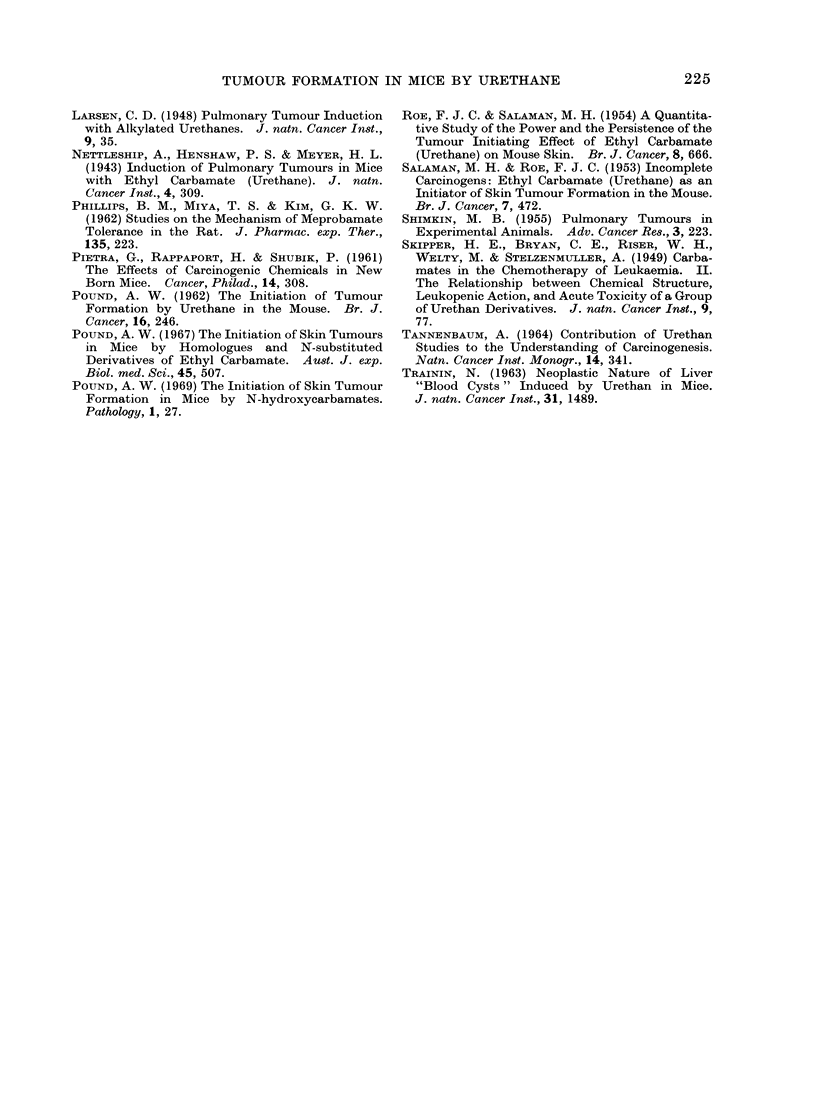# Tumour Formation in Mice by Urethane Administered With Related Carbamates

**DOI:** 10.1038/bjc.1972.29

**Published:** 1972-06

**Authors:** A. W. Pound

## Abstract

A tumour initiating dose of ethyl carbamate was administered to mice by subcutaneous injection together with a dose of one of the homologous esters or an ethyl N-alkyl derivative. The homologues used were the methyl, *n*-propyl and *n*-butyl esters, and the derivatives were the N-methyl, N-ethyl and N-*n*-propyl ethyl esters. The mice were then given promoting treatment with croton oil for 28 weeks.

Neither the homologous esters nor the N-substituted derivatives of ethyl carbamate had any influence on the yield of tumours in the skin, lung, or liver. However, increasing the dose of ethyl carbamate increased the yields of tumours.


*Br. J. Cancer* (1972) **26**, 216

### TUMOUR FORMATION IN MICE BY URETHANE ADMINISTERED WITH RELATED CARBAMATES

A. W. POUND

*Department of Pathology, University of Queensland, Brisbane, Australia*

Received for publication December 1971

**Summary.—A tumour initiating dose of ethyl carbamate was administered to mice by subcutaneous injection together with a dose of one of the homologous esters or an ethyl N-alkyl derivative. The homologues used were the methyl, *n*-propyl and *n*-butyl esters, and the derivatives were the N-methyl, N-ethyl and N-*n*-propyl ethyl esters. The mice were then given promoting treatment with croton oil for 28 weeks.**

**Neither the homologous esters nor the N-substituted derivatives of ethyl carbamate had any influence on the yield of tumours in the skin, lung, or liver. However, increasing the dose of ethyl carbamate increased the yields of tumours.**

THE administration of ethyl carbamate (urethane) to mice by any route leads to the production of tumours in a variety of tissues (Tannenbaum, 1964). Adenomata of the lung (Nettleship *et al.*, 1943; Shimkin, 1955) are very common. Lymphomata with or without leukaemia (Pietra *et al.*, 1961), haemangiomata of the liver (Trainin, 1963), and hepatomata are less commonly found; tumours of lacrimal gland, skin and of other sites are observed infrequently and only after a long latent period. For skin its carcinogenic action is mainly that of an initiator, that is, tumours usually appear only when the skin is subsequently painted repeatedly with a promoting agent such as croton oil (Graffi *et al.*, 1953; Salaman and Roe, 1953; Berenblum and Haran, 1955).

The tumour initiating property of carbamates for skin appears to be limited to the ethyl ester and a few of its N-substituted derivatives (Berenblum *et al.*, 1959*b*; Pound, 1967, 1969). The tumour producing property for lung also appears to be restricted to ethyl, ethyl N-methyl and N-hydroxy carbamates although propyl carbamate was considered to have a doubtful effect (Larsen, 1947, 1948; Berenblum *et al.*, 1959*b*).

However, because of the common chemical structure it might be thought that the homologues, or the N-substituted derivatives, of ethyl carbamate might affect or be affected by similar metabolic systems within the cells of animals injected with them. Some related compounds appear to produce similar abnormalities of the chromosomes during mitosis in the cells of animals, some eggs and plants (Cornman, 1954; Boyland *et al.*, 1965), and lead to a similar depression of the white cell counts in mice (Skipper *et al.*, 1949).

The injection of homologues or derivatives of ethyl carbamate at the same time as the ethyl ester might therefore modify the tumour producing properties of the latter. Garcia (1963) reported that injection of butyl carbamate at the same time as the ethyl ester reduced the number of tumours initiated in the skin. The experiments reported in this paper were carried out to examine this proposition.

### MATERIALS AND METHODS

### *Mice*

Random bred male mice of the strain " Hall " (Pound, 1962), about 7 weeks of age and weighing 26 ± 1 g at the beginning of the experiments, were used. The animals were divided into groups by random selection and were housed in stainless steel compartments each containing 10 mice with a bed of coarse sawdust that was changed weekly. The animals were fed the standard diet used previously and supplied with water *ad libitum*. The mouse room was kept at a temperature of 22°C. The hair on the skin of the back was clipped with electric clippers an hour or two before application of the tumour promoting agents to the skin.

*Chemicals*

The chemicals used were as follows: ethyl carbamate (British Drug Houses analytical reagent); methyl, isopropyl, *n*-butyl, ethyl N-methyl, ethyl N-ethyl and ethyl N-*n*-propyl carbamates (K. and K. Laboratories Inc., New York); *n*-propyl carbamate (Eastman Organic Chemicals, Rochester, New York); acetone (Anax analytical reagent). Croton oil (A) used in Experiment I was a sample from Stafford Allen and Sons, London, used previously. Croton oil (B) used in Experiments II and III was prepared by extraction of the seed of *Croton tiglium* (*L*) with methanol in an atmosphere of nitrogen.

The carbamates were administered in 0·75 ml of saline as an injection into the subcutaneous tissue between the scapulae. Solid carbamates were kept in solution by warming when necessary. When the liquid carbamates were not completely soluble 1% serum (human) was included in the solution and reasonably stable emulsions then formed on shaking.

*Tumour initiating experiments*

Groups of 40 mice were injected with 25 mg of urethane (0·432 $LD_{50}$) and at the same time with one of the homologues or an additional amount of urethane. Two groups of mice were injected with 25 mg of urethane alone for each experiment, one before and the other after the main series of injections. Experiment I was commenced in April 1963; Experiments II and III were commenced 3 years later and the mice were randomized between these 2 experiments.

Thus the mice were injected with 0·432 $LD_{50}$ urethane in the controls. This was increased to 0·540, 0·650 and 0·864 $LD_{50}$ by the additional amount of urethane in Experiments I, II and III respectively. The original plan was to inject the other carbamates in molecular equivalent amounts to the additional doses of urethane, but these doses were too lethal. The additional dose in each experiment was therefore selected at an arbitrary figure, standard for each experiment as set out in the tables below.

In Experiment I the doses of the homologues were as shown in Table II. From the 7th day after injection of the carbamates, the mice were painted over the whole area of the skin of the back with 0·25 ml of a 0·5% solution of croton oil (A) in acetone once each week for 20 weeks. The time of appearance of the first tumour was noted and the numbers of tumours present recorded on charts at the 14th and 22nd weeks. The mice were then discarded without autopsy.

In Experiments II and III the additional doses of urethane were 12·5 mg and 25 mg respectively. The doses of homologues were increased in Experiment II and again in Experiment III as shown in Table III. From the 7th day after injection of the carbamates the mice were given an application of 0·25 ml of a 0·075% solution of croton oil (B) in acetone over the whole area of the skin of the back once weekly for 28 weeks. The time of appearance of the first tumour was noted and the number of tumours present on the painted area were recorded on charts at 12, 16, 20, 28, and 36 weeks. Animals that died before this time were discarded without autopsy. After the 40th week obviously sick animals were killed and with the remaining animals, which were all killed at 50 weeks, subjected to autopsy. A few animals died undetected early enough to be suitable for autopsy. Skin lesions that were considered to be malignant or doubtfully malignant were confirmed by histological examination, but it was not practical to section all lesions. The number of mice with lung adenomata and the number of lesions were counted. Haemangiomata and hepatomata in the liver were counted and the diagnosis confirmed histologically. The number of mice with leukaemia was noted; enlargement of the spleen and/or the thymus was accepted as evidence of leukaemia, but in occasional cases blood smears or histological sections were examined.

### RESULTS

### *General Toxic and Narcotic Effects*

The depth of narcosis produced by the basal dose of ethyl carbamate was increased, as expected, by the additional

TABLE I.—*Toxic Effects of Combined Injection of Urethane with Another Carbamate*

| Mice injected with 25 mg urethane together with | Experiment I | | | | | Experiment II | | | | | Experiment III | | | | |
|---|---|---|---|---|---|---|---|---|---|---|---|---|---|---|---|
| | mg | Fraction of $LD_{50}$ | Total fraction $LD_{50}$ | SE | SF | mg | Fraction of $LD_{50}$ | Total fraction $LD_{50}$ | SE | SF | mg | Fraction of $LD_{50}$ | Total fraction $LD_{50}$ | SE | SF |
| — | — | — | 0·43 | 40 | 40 | — | — | 0·43 | 40 | 40 | — | 0·43 | 0·43 | 40 | 40 |
| Methyl carbamate | 5·0 | 0·04 | 0·47 | 40 | 40 | 10·0 | 0·09 | 0·51 | 40 | 40 | 20·0 | 0·17 | 0·60 | 40 | 40 |
| *n*-Propyl carbamate | 5·0 | 0·15 | 0·58 | 40 | 40 | 10·0 | 0·30 | 0·73 | 38 | 39 | 15·0 | 0·44 | 0·88 | 34 | 24 |
| *i*-Propyl carbamate | 5·0 | 0·15 | 0·58 | 40 | 40 | 10·0 | 0·30 | 0·73 | 38 | 39 | 15·0 | 0·44 | 0·87 | 34 | 27 |
| Butyl carbamate | 5·0 | 0·36 | 0·79 | 36 | 37 | 10·0 | 0·71 | 1·14 | 8 | 16 | 15·0 | 1·07 | 1·50 | 0 | 0 |
| Ethyl N-methyl carbamate | 5·0 | 0·14 | 0·57 | 40 | 40 | 10·0 | 0·28 | 0·71 | 38 | 37 | 15·0 | 0·42 | 0·85 | 34 | 19 |
| Ethyl N-ethyl carbamate | 5·0 | 0·22 | 0·66 | 40 | 40 | 10·0 | 0·45 | 0·88 | 34 | 39 | 15·0 | 0·67 | 1·10 | 8 | 8 |
| Ethyl N-*n*-propyl carbamate | 5·0 | 0·36 | 0·79 | 36 | 40 | 10·0 | 0·71 | 1·14 | 8 | 26 | 15·0 | 1·07 | 1·50 | 0 | 3 |
| Ethyl carbamate | 6·252 | 0·11 | 0·55 | 40 | 40 | 12·5 | 0·22 | 0·65 | 40 | 38 | 25·0 | 0·43 | 0·86 | 33 | 33 |
| — | — | — | 0·43 | 40 | 40 | — | — | 0·43 | 40 | 40 | — | 0·43 | 0·43 | 40 | 40 |

SE = Survivors expected; SF = Survivors found.

doses of urethane or other carbamate, and some animals died within 48 hours from this cause. Table I sets out the dosage schedules in the 3 experiments. The basic dose of ethyl carbamate in each experiment was 25 mg, *i.e.* 0·432 $LD_{50}$. The dosage of the additional carbamate for each group is shown as a fraction of its $LD_{50}$ as previously determined (Pound, 1967). The sums of the fractions of the $LD_{50}$ are also shown. The number of survivors expected for the total fraction of the $LD_{50}$ does not differ significantly from the number of survivors actually found. The narcotic and toxic properties of the several carbamates are therefore additive when the doses are considered as fractions of the $LD_{50}$.

Apart from the early deaths due to narcosis, the death rates in the 3 experiments and in all groups of each experiment do not differ significantly.

### *Classification of Skin Tumours*

The skin tumours that arise after initiation by urethane, followed by promoting treatment with croton oil, behave in a variety of ways that enable them to be classified for the purpose of this paper as benign papillomata, doubtfully malignant lesions or carcinomata.

#### *Benign papillomata*

These constitute the majority of growths. The lesions are first identified when about 1 mm in diameter. A number of lesions grow for a time and regress during the treatment with croton oil, but some continue to grow and may reach a size ranging from 3 to 10 mm diameter. Growth may continue even after cessation of the croton oil treatment, and further lesions may reach detectable size. As the lesions age after cessation of the promoting treatment a further proportion undergo regression.

Histologically these lesions are typical papillomata clothed by hyperplastic stratified squamous epithelium of regular character with a varying amount of keratin formation, sometimes abundant. As the lesions age they become less vascular and form more keratin. Some finally atrophy and disappear, but occasional lesions become necrotic and are sloughed off.

#### *Doubtfully malignant lesions*

A proportion of the lesions commence to grow in a similar manner to benign papillomata but grow progressively and may reach a considerable size, *e.g.* 12–15 mm in diameter. These lesions remain vascular, become fleshy, and may differ considerably in appearance from the benign lesions. They may have a broad pedicle or sit well into the dermis.

Histologically these lesions have a papillary structure clothed by a deeply staining active squamous epithelium with many mitoses, and usually form much keratin. The epithelium shows varying degrees of invasion at the base, into the dermis, or even into the underlying fat.

Lesions of this type rarely regress in the period of observation. In this experiment an occasional pedunculated lesion sloughed off probably following mechanical trauma during clipping. In one instance in which this occurred, a squamous cell carcinoma developed later at the site. The impression has been formed that a substantial proportion of these lesions would become carcinoma if their growth had been allowed to continue.

#### *Carcinomata*

A further proportion of the lesions become frank carcinoma during the period of observation. Histologically these vary from moderately well differentiated squamous cell carcinomata to anaplastic spindle-celled growths, with invasion of the deeper tissue, the *muscularis panniculous carnosus*, and even the body musculature.

### *Tumour Yields*

The number of mice with tumours of the skin and the number of skin tumours in each group at the 14th and 22nd week

TABLE II.—*Incidence of Skin Tumours in Mice Injected with Urethane Together with a Homologous Ester or an N-substituted Derivative Experiment I*

| Mice injected with 25 mg urethane together with | mg | Survivors 24 hours after injection | Surviving mice 14 weeks: Number of mice | Mice with tumours | Number of tumours | Surviving mice 22 weeks: Number of mice | Mice with tumours | Number of tumours |
|---|---|---|---|---|---|---|---|---|
| — | — | 40 | 38 | 2 | 3 | 38 | 7 | 10 (2) |
| Methyl carbamate | 5·0 | 40 | 36 | 3 | 5 | 33 | 7 | 10 (1) |
| *n*-Propyl carbamate | 5·0 | 40 | 38 | 2 | 2 | 37 | 7 | 9 |
| *i*-Propyl carbamate | 5·0 | 40 | 34 | 2 | 2 | 33 | 4 | 7 |
| *n*-Butyl carbamate | 5·0 | 37 | 37 | 1 | 2 | 33 | 8 | 13 (2) |
| Ethyl N-methyl carbamate | 5·0 | 40 | 35 | 2 | 2 | 35 | 6 | 7 |
| Ethyl N-ethyl carbamate | 5·0 | 40 | 39 | 3 | 4 | 39 | 8 | 14 (1) |
| Ethyl N-*n*-propyl carbamate | 5·0 | 40 | 39 | 2 | 3 | 38 | 6 | 10 (1) |
| — | — | 40 | 38 | 2 | 3 | 37 | 7 | 11 (1) |
| Ethyl carbamate | 6·5 | 40 | 36 | 2 | 4 | 36 | 8 | 15 (2) |

Figures in brackets are the numbers of the tumours that were malignant of Experiment I are set out in Table II. Similar parameters after the 12th, 20th and 36th weeks of Experiments II and III are set out in Table III. Table IV sets out the data from autopsy of animals that died after the 40th week or were killed at 50 weeks. Skin tumours were classified as above from the clinical behaviour *in vivo*, or from sections of doubtful lesions taken at autopsy.

Comparison of the tumour yields in the skin of the control groups injected with 25 mg of urethane in Experiments I, II, and III after approximately 20 weeks' promoting treatment shows that the number of mice with tumours, and the total number of tumours in the surviving mice, are approximately the same in Experiments II and III but that the yield of tumours is significantly greater in these experiments than in Experiment I (for the number of tumours per surviving mice $\chi^2 = 9{\cdot}22$, 2 d.f., $P < 0{\cdot}01$). This is probably due to a greater promoting efficiency of the croton oil (B) used in Experiments II and III which was effective as a 0·075% solution in acetone as compared with the 0·5% solution of croton oil (A) used in Experiment I. In both instances the concentrations were about the maximum that could be used without producing ulceration of the skin.

The results of Experiment I (Table II) show no significant variation in survival rate between the several groups ($\chi^2 = 2{\cdot}5$, 9 d.f., N.S.). Further, there is no significant variation between the proportion of surviving mice with tumours ($\chi^2 = 4{\cdot}6$, 9 d.f., N.S.) or the total number of tumours in the surviving mice ($\chi^2 = 4{\cdot}6$, 9 d.f., N.S.) in the various groups. Even the additional dose of urethane in Group 10 did not produce a statistically significant increase in the tumour yield.

The results of Experiments II and III (Tables III and IV) may be considered together since the results show no significant variation in the tumour yields in the control groups injected with 25 mg of urethane. Apart from the early deaths due to toxic effects of the carbamates, the death rates do not differ in the 2 experiments.

If the Groups 9 in Experiments II and III are excluded, there is no significant variation in the number of mice with skin tumours or in the number of tumours in the surviving mice at 12, 20, 36, and 50 weeks after the administration of the urethane. There is no significant variation in these groups in the proportion of surviving mice with adenomata in the lungs, nor in the number of adenomata. The number of tumours in the liver, haemangiomata and hepatomata, and the number of mice with leukaemia do not vary significantly between the groups.

On the other hand, the number of tumours in the surviving mice is significantly greater in Group 9 of each

TABLE III.—*Incidence of Skin Tumours in Mice Injected with Urethane Together with a Homologous Ester of a N-substituted Derivative Experiments II and III*

| Experiment | Group | Injected with 25 mg urethane: Together with | mg | Survivors 24 hours after injection | Surviving mice 12 weeks: No. of mice | 12 weeks, Mice with tumours: No. of mice | 12 weeks, Mice with tumours: No. of tumours | Surviving mice 20 weeks: No. of mice | 20 weeks, Mice with tumours: No. of mice | 20 weeks, Mice with tumours: No. of tumours | Surviving mice 36 weeks: No. of mice | 36 weeks, Mice with tumours: No. of mice | No. of tumours: Total No. of tumours | No. of tumours: Prob. malignant | No. of tumours: Malignant |
|---|---|---|---|---|---|---|---|---|---|---|---|---|---|---|---|
| II | 1 | — | — | 40 | 40 | 3 | 4 | 33 | 14 | 23 | 34 | 19 | 42 | 2 | 2 |
| | 2 | Methyl carbamate | 10·0 | 40 | 39 | 2 | 3 | 39 | 11 | 18 | 36 | 16 | 47 | 2 | 3 |
| | 3 | *n*-Propyl carbamate | 10·0 | 39 | 39 | 2 | 2 | 39 | 9 | 15 | 39 | 19 | 30 | 2 | 2 |
| | 4 | *i*-Propyl carbamate | 10·0 | 39 | 37 | 3 | 3 | 35 | 12 | 23 | 35 | 20 | 49 | 2 | 3 |
| | 5 | *n*-Butyl carbamate | 10·0 | 16 | 13 | 1 | 1 | 11 | 5 | 5 | 9 | 5 | 8 | 2 | 0 |
| | 6 | Ethyl-N-methyl carbamate | 10·0 | 37 | 34 | 2 | 2 | 30 | 9 | 17 | 28 | 13 | 32 | 3 | 1 |
| | 7 | Ethyl N-ethyl carbamate | 10·0 | 39 | 37 | 3 | 3 | 35 | 10 | 20 | 31 | 13 | 36 | 3 | 2 |
| | 8 | Ethyl-N-*n* propyl carbamate | 10·0 | 26 | 20 | 3 | 4 | 18 | 10 | 20 | 15 | 13 | 27 | 3 | 2 |
| | 9 | Ethyl carbamate | 12·5 | 38 | 37 | 5 | 7 | 33 | 14 | 34 | 31 | 19 | 56 | 9 | 5 |
| | 10 | — | — | 40 | 39 | 3 | 5 | 37 | 13 | 18 | 35 | 17 | 33 | 4 | 3 |
| III | 1 | — | — | 40 | 40 | 2 | 4 | 36 | 11 | 18 | 33 | 19 | 43 | 4 | 0 |
| | 2 | Methyl carbamate | 20 | 40 | 38 | 5 | 5 | 40 | 15 | 22 | 36 | 18 | 38 | 3 | 3 |
| | 3 | *n*-Propyl carbamate | 15 | 24 | 24 | 2 | 2 | 23 | 9 | 14 | 21 | 13 | 33 | 1 | 1 |
| | 4 | *i*-Propyl carbamate | 15 | 27 | 22 | 2 | 3 | 21 | 5 | 6 | 17 | 11 | 23 | 1 | 1 |
| | 5 | *n*-Butyl carbamate | 15 | 0 | — | — | — | — | — | — | — | — | — | — | — |
| | 6 | Ethyl-N-methyl carbamate | 15 | 19 | 15 | 2 | 3 | 13 | 6 | 10 | 11 | 6 | 16 | 2 | 1 |
| | 7 | Ethyl-N-ethyl carbamate | 15 | 8 | 7 | 0 | 0 | 7 | 5 | 8 | 6 | 5 | 15 | 1 | 0 |
| | 8 | Ethyl N-*n* propyl carbamate | 15 | 3 | — | — | — | — | — | — | — | — | — | — | — |
| | 9 | Ethyl carbamate | 25 | 33 | 31 | 4 | 9 | 30 | 16 | 48 | 28 | 21 | 87 | 10 | 9 |
| | 10 | — | — | 40 | 38 | 4 | 8 | 35 | 11 | 25 | 33 | 17 | 36 | 5 | 3 |

TABLE IV.—*Incidence of Tumours of Skin, Lung, and Liver, and of Leukaemia in Mice Surviving* 40–50 *Weeks Experiments II and III*

| | | | Autopsies of mice 40th to 56th weeks | | | | | | | | |
|---|---|---|---|---|---|---|---|---|---|---|---|
| | | | Mice with skin tumours | | | | Lung adenomata | | Liver tumours | | |
| | | | | Numbers of tumours | | | | | | | |
| Experiment | Group | Total number of mice | Number of mice | Benign | Doubtful | Malignant | Number of mice | Number tumours | Hepatoma | Haeman-gioma | Mice with leukaemia |
| II | 1 | 33 (3) | 16 | 17 | 3 | 4 | 15 | 41 | 2 | 2 | 4 |
| | 2 | 34 (4) | 16 | 20 | 5 | 5 | 18 | 45 | 1 | 3 | 4 |
| | 3 | 39 | 15 | 14 | 6 | 4 | 16 | 33 | 0 | 6 | 5 |
| | 4 | 24 | 10 | 11 | 3 | 2 | 10 | 25 | 2 | 2 | 3 |
| | 5 | 9 | 6 | 5 | 5 | 0 | 4 | 9 | 0 | 1 | 2 |
| | 6 | 28 | 12 | 8 | 2 | 2 | 13 | 29 | 2 | 5 | 3 |
| | 7 | 29 | 14 | 14 | 5 | 2 | 15 | 35 | 3 | 4 | 4 |
| | 8 | 16 | 12 | 20 | 4 | 2 | 7 | 16 | 1 | 1 | 3 |
| | 9 | 30 (1) | 17 | 26 | 9 | 7 | 19 | 65 | 2 | 5 | 5 |
| | 10 | 34 | 16 | 17 | 4 | 6 | 16 | 44 | 3 | 2 | 5 |
| III | 1 | 31 (2) | 14 | 22 | 4 | 2 | 19 | 34 | 1 | 3 | 4 |
| | 2 | 35 (1) | 16 | 20 | 4 | 3 | 14 | 35 | 0 | 2 | 5 |
| | 3 | 21 | 10 | 7 | 4 | 2 | 7 | 16 | 2 | 5 | 3 |
| | 4 | 17 | 6 | 9 | 2 | 2 | 5 | 11 | 3 | 1 | 4 |
| | 5 | — | — | — | — | — | — | — | — | — | — |
| | 6 | 11 | 6 | 9 | 3 | 2 | 7 | 16 | 2 | 2 | 3 |
| | 7 | 6 | 4 | 6 | 1 | 1 | 3 | 11 | 1 | 0 | 0 |
| | 8 | — | — | — | — | — | — | — | — | — | — |
| | 9 | 27 (2) | 21 | 33 | 20 | 9 | 25 | 130 | 2 | 8 | 7 |
| | 10 | 30 (2) | 11 | 14 | 5 | 3 | 21 | 34 | 1 | 3 | 5 |

experiment, which received the additional dose of urethane (Experiment II, $\chi^2 = 9 \cdot 4$ 1 d.f., $P < 0 \cdot 005$ at 36th week; Experiment III, $\chi^2 = 49$ 1 d.f., $P < 0 \cdot 001$ at 36th week). Further, the tumour yield is greater in the mice in Group 9 of Experiment III, which had a dose of 50 mg of urethane, than in Group 9 of Experiment II which had 37·5 mg. The increased tumour yields in Group 9 of Experiments II and III are significant at the 12th, 20th, 36th, and 50th weeks.

The proportion of the surviving mice with lung tumours and the number of lung adenomata are also increased in Groups 9 of Experiments II and III. The number of lung adenomata is significantly greater in Group 9 of Experiment III than in Group 9 of Experiment II, in conformity with the larger dose of urethane.

The number of liver tumours and the number of mice with leukaemia do not vary significantly in any group but the numbers are too small to allow any valid conclusion.

It is evident that the number of tumours increases steadily from the time of appearance of the first tumours at about the 8th week for about 36 weeks and thereafter declines. Since the applications of croton oil were discontinued after 28 weeks, tumours must continue to develop for a time after cessation of the promoting treatment before the number regressing becomes greater than the number of new ones appearing. In these experiments many tumours eventually regressed. On the other hand, the number of malignant tumours increased steadily from the time they were first recognized.

### DISCUSSION

It is relevant to the present issue that, when a given dose of urethane was injected together with graded doses of one or other of its homologues or N-substituted derivatives, the narcotic potencies of the combined injections were such as to be expected if the toxicity of the compounds is additive in accordance with Ferguson's rule (Ferguson, 1939).

When the mice were given a fixed tumour initiating dose of ethyl carbamate, the simultaneous injection of one of the homologous methyl, propyl, and ethyl carbamates, or of the related ethyl N-methyl, N-ethyl, or N-propyl carbamates, did not produce any significant change in the tumour yield in any tissue. The proportion of mice that developed skin tumours and the total number of skin tumours in the surviving mice was unaltered at any stage after promoting treatment was commenced, that is, the rate of production of tumours and the rate of regression was unaltered and, moreover, the number of malignant tumours was also unchanged.

On the other hand, an increase in the dose of ethyl carbamate increased the number of lung tumours, the number of tumours in the liver, and perhaps the incidence of leukaemia. It produced an increase in the yield of skin tumours at all times after promoting treatment was commenced, and in the proportion of skin tumours that were malignant. A similar increased yield of tumours with increasing dose of urethane has been found by others (Berenblum and Haran-Ghera, 1957; Roe and Salaman, 1954).

The failure of the homologous alkyl carbamates to increase the tumour yield is consistent with previous work, suggesting that these compounds have very little, if any, potency as tumour producing agents in mice for skin and lung (Larsen, 1947, 1948; Berenblum *et al.*, 1959*b*; Pound, 1967 and unpublished data). The fact that they, as well as the N-substituted derivatives of ethyl carbamate, had *no effect* on the tumour yield substantiates the view that the biochemical events resulting in the production of tumours by urethane are not related to those cellular processes that result in narcosis. This view was formerly based on observations that the tumour producing properties and the narcotic properties of carbamates depend on different chemical features of the molecule (Berenblum *et al.*, 1959*b*; Pound, 1967, 1969), and that the administration of lysergic acid diethylamide (LSD-25) at the same time as urethane obviates the narcosis but does not influence the tumour yields (Berenblum *et al.*, 1959*a*).

Ethyl-N-methyl carbamate, however, is more than half as potent as ethyl carbamate, while ethyl-N-ethyl and ethyl-N-propyl carbamates are progressively less active in their tumour initiating property for mouse skin and tumour producing property in mouse lung (Berenblum *et al.*, 1959*b*; Pound, 1967 and unpublished data). The higher doses of ethyl-N-methyl carbamate would probably be effective if given alone and perhaps should be expected to lead to increased tumour yields when given together with ethyl carbamate if the tumour producing effects were simply additive in the same way as an addition to the dose of urethane, particularly since they are chemically closely related and probably act through the same metabolic pathways. Nevertheless, no significant increase in tumour yields was found. Perhaps further information on this point might be obtained. However, the carcinogenic potency of carbamates appears to be fairly specific to the ethyl esters with not more than one hydrogen substituted on the amide nitrogen (Pound, 1967, 1969), suggesting the existence of a specific metabolic pathway to an active intermediate. The present result suggests that the unsubstituted ethyl carbamate is metabolized to the active carcinogen in preference to the N-substituted compounds when both are present in an animal.

Garcia (1963) reported that the administration of butyl carbamate at the same time as the ethyl ester reduced the number of skin tumours obtained after subsequent promoting treatment with croton oil. The discrepancy with the present results may be related to the fact that he administered the compounds as a series of small injections, over a period which appears to have been about 23 days in one group, although the actual dosage schedules were not set out. These conditions are likely to induce the formation of enzymes that metabolize the compounds, as is known to occur in the case of ethyl carbamate (Conney, 1965) and the dicarbamate meprobamate (Phillips *et al.*, 1962), so that less is available for carcinogenic action. A report that the administration of ethyl carbamate as a course of injections over several days resulted in the initiation of fewer tumours than if it had been given as a single large dose (Berenblum and Haran-Ghera, 1957) possibly reflects a similar phenomenon.

This work was supported by grants from the Queensland Cancer Fund and the Mayne Bequest Fund of the University of Queensland.

### REFERENCES

Berenblum, I. & Haran, Nechama (1955) The Initiating Action of Ethyl Carbamate (Urethane) on Mouse Skin. *Br. J. Cancer*, **9**, 453.

Berenblum, I. & Haran-Ghera, Nechama (1957) A Quantitative Study of the Systemic Initiating Action of Urethane (Ethyl Carbamate) in Mouse Skin Carcinogenesis. *Br. J. Cancer*, **11**, 77.

Berenblum, I., Blum, B. & Trainin, N. (1959*a*) Failure of the Urethane Antagonist Lysergic Acid-diethylamide (LSD-25) to Inhibit Lung Carcinogenesis or the Initiating Phase of Skin Carcinogenesis in Mice. *Biochem. Pharmac.*, **2**, 197.

Berenblum, I., Ben Ishai, D., Haran-Ghera, N., Lapidot, A., Simon, E. & Trainin, N. (1959*b*) Skin Initiating Action and Lung Carcinogenesis by Derivatives of Urethane (Ethyl Carbamate) and Related Components. *Biochem. Pharmac.*, **2**, 168.

Boyland, E., Nery, R. & Peggie, K. S. (1965) The Induction of Chromosome Aberrations in *Vicia Faba* Root Meristems by N-hydroxyurethane and Related Components. *Br. J. Cancer*, **19**, 878.

Conney, A. H. (1965) *Drugs and Enzymes*. Proc. 2nd Internat. Pharmacol. Meeting. Ed. B. B. Brodie & J. R. Gillette. New York: Pergamon. p. 277.

Cornman, I. (1954) The Properties of Urethane Considered in Relation to its Action on Mitosis. *Int. Rev. Cytol.*, **3**, 113.

Ferguson, J. (1939) The Use of Chemical Potentials as Indices of Toxicity. *Proc. R. Soc. B*, **172**, 387.

Garcia, H. (1963) Inhibition of Tumorigenic Action of Urethane by Butyl Carbamate. *Biologica, Santiago*, **34**, 11.

Graffi, A., Vlamynck, E., Hoffman, F. & Schulz, I. (1953) Untersuchungen über die geschwulstauslösende Wirkung verschiedener chemischer Stoffe in der Kombination mit Crotonöl. *Arch. Geschwulstforsch*, **5**, 110.

Larsen, C. D. (1947) Evaluation of the Carcinogenicity of a Series of Esters of Carbamic Acid. *J. natn. Cancer Inst.*, **8**, 99.

LARSEN, C. D. (1948) Pulmonary Tumour Induction with Alkylated Urethanes. *J. natn. Cancer Inst.*, **9**, 35.
NETTLESHIP, A., HENSHAW, P. S. & MEYER, H. L. (1943) Induction of Pulmonary Tumours in Mice with Ethyl Carbamate (Urethane). *J. natn. Cancer Inst.*, **4**, 309.
PHILLIPS, B. M., MIYA, T. S. & KIM, G. K. W. (1962) Studies on the Mechanism of Meprobamate Tolerance in the Rat. *J. Pharmac. exp. Ther.*, **135**, 223.
PIETRA, G., RAPPAPORT, H. & SHUBIK, P. (1961) The Effects of Carcinogenic Chemicals in New Born Mice. *Cancer, Philad.*, **14**, 308.
POUND, A. W. (1962) The Initiation of Tumour Formation by Urethane in the Mouse. *Br. J. Cancer*, **16**, 246.
POUND, A. W. (1967) The Initiation of Skin Tumours in Mice by Homologues and N-substituted Derivatives of Ethyl Carbamate. *Aust. J. exp. Biol. med. Sci.*, **45**, 507.
POUND, A. W. (1969) The Initiation of Skin Tumour Formation in Mice by N-hydroxycarbamates. *Pathology*, **1**, 27.
ROE, F. J. C. & SALAMAN, M. H. (1954) A Quantitative Study of the Power and the Persistence of the Tumour Initiating Effect of Ethyl Carbamate (Urethane) on Mouse Skin. *Br. J. Cancer*, **8**, 666.
SALAMAN, M. H. & ROE, F. J. C. (1953) Incomplete Carcinogens: Ethyl Carbamate (Urethane) as an Initiator of Skin Tumour Formation in the Mouse. *Br. J. Cancer*, **7**, 472.
SHIMKIN, M. B. (1955) Pulmonary Tumours in Experimental Animals. *Adv. Cancer Res.*, **3**, 223.
SKIPPER, H. E., BRYAN, C. E., RISER, W. H., WELTY, M. & STELZENMULLER, A. (1949) Carbamates in the Chemotherapy of Leukaemia. II. The Relationship between Chemical Structure, Leukopenic Action, and Acute Toxicity of a Group of Urethan Derivatives. *J. natn. Cancer Inst.*, **9**, 77.
TANNENBAUM, A. (1964) Contribution of Urethan Studies to the Understanding of Carcinogenesis. *Natn. Cancer Inst. Monogr.*, **14**, 341.
TRAININ, N. (1963) Neoplastic Nature of Liver "Blood Cysts" Induced by Urethan in Mice. *J. natn. Cancer Inst.*, **31**, 1489.